# A high-throughput method for the detection of homoeologous gene deletions in hexaploid wheat

**DOI:** 10.1186/1471-2229-10-264

**Published:** 2010-11-29

**Authors:** Timothy L Fitzgerald, Kemal Kazan, Zhongyi Li, Matthew K Morell, John M Manners

**Affiliations:** 1 CSIRO Plant Industry, 306 Carmody Road, St Lucia, QLD 4067, Australia; 2 CSIRO Plant Industry, GPO Box 1600, Canberra ACT 2601, Australia; 3 CSIRO Food Futures National Research Flagship, PO Box 93, North Ryde 1670, NSW, Australia

## Abstract

**Background:**

Mutational inactivation of plant genes is an essential tool in gene function studies. Plants with inactivated or deleted genes may also be exploited for crop improvement if such mutations/deletions produce a desirable agronomical and/or quality phenotype. However, the use of mutational gene inactivation/deletion has been impeded in polyploid plant species by genetic redundancy, as polyploids contain multiple copies of the same genes (homoeologous genes) encoded by each of the ancestral genomes. Similar to many other crop plants, bread wheat (*Triticum aestivum *L.) is polyploid; specifically allohexaploid possessing three progenitor genomes designated as 'A', 'B', and 'D'. Recently modified TILLING protocols have been developed specifically for mutation detection in wheat. Whilst extremely powerful in detecting single nucleotide changes and small deletions, these methods are not suitable for detecting whole gene deletions. Therefore, high-throughput methods for screening of candidate homoeologous gene deletions are needed for application to wheat populations generated by the use of certain mutagenic agents (e.g. heavy ion irradiation) that frequently generate whole-gene deletions.

**Results:**

To facilitate the screening for specific homoeologous gene deletions in hexaploid wheat, we have developed a TaqMan qPCR-based method that allows high-throughput detection of deletions in homoeologous copies of any gene of interest, provided that sufficient polymorphism (as little as a single nucleotide difference) amongst homoeologues exists for specific probe design. We used this method to identify deletions of individual *TaPFT1 *homoeologues, a wheat orthologue of the disease susceptibility and flowering regulatory gene *PFT1 *in Arabidopsis. This method was applied to wheat nullisomic-tetrasomic lines as well as other chromosomal deletion lines to locate the *TaPFT1 *gene to the long arm of chromosome 5. By screening of individual DNA samples from 4500 M2 mutant wheat lines generated by heavy ion irradiation, we detected multiple mutants with deletions of each *TaPFT1 *homoeologue, and confirmed these deletions using a CAPS method. We have subsequently designed, optimized, and applied this method for the screening of homoeologous deletions of three additional wheat genes putatively involved in plant disease resistance.

**Conclusions:**

We have developed a method for automated, high-throughput screening to identify deletions of individual homoeologues of a wheat gene. This method is also potentially applicable to other polyploidy plants.

## Background

Identification of plant gene function is important not only to better understand how plants grow and develop, but also for potential exploitation of this information for molecular and/or traditional crop breeding. However, extensive gene duplication and polyploidy events during evolution mean that plants carry multiple copies of the same gene as well as large numbers of closely-related genes [1]. Therefore, for gene function studies, it is important to determine whether different copies of the same or related genes are functionally redundant or whether polymorphisms have been selected through evolution and led to functional specialisation [2,3]. Reverse genetics approaches to answering these questions range from the development of transgenic plants where individual gene copies are silenced [4,5] to the identification of mutants where gene copies have been either modified, inactivated by sequence alterations [6-8], or completely deleted [9]. For the model plant Arabidopsis, the availability of large numbers of T-DNA (or transposon) insertion lines makes it increasingly likely that knock-out insertion mutants can be identified for specific copies of selected closely-related genes to test for redundancy or a specific function [10]. In many other plants, including most of the important crop species, this task is not so straightforward and new technical approaches for the identification of mutations in specific gene copies are needed [11].

Mutant populations of many crop species including wheat have been developed through chemical mutagenesis, and mutations in a range of target genes have been identified in such populations using 'Targeting Induced Local Lesions IN Genomes' (TILLING) [12,13] techniques. In TILLING, a gene region is amplified from pools of genomic DNA, amplified fragments allowed to re-anneal, and mutations detected by testing for mismatches between wild type and mutant heteroduplexes [13,14]. This technique is capable of detecting the point mutations, re-arrangements, and small deletions [6] that chemical mutagens predominantly produce [13], but it is not directly applicable to screening for whole-gene deletions. An additional technical difficulty in applying TILLING to polyploid crop species such as wheat is that TILLING requires independent amplification of homoeologous gene copies to prevent heteroduplexes forming between these closely-related sequences, as this can confound the subsequent data analysis. Therefore, the development of new high-throughput methods that allow simultaneous detection of mutations in homoeologous genes in a single assay, as has recently been reported [15], is certainly highly desirable for functional genomics studies in wheat and other polyploidy crop species.

Recently, physical mutagenesis such as heavy ion irradiation (HII) has been used to generate mutant populations of model, crop and ornamental plant species [16-18]. In contrast to chemical mutagenesis (e.g. ethyl methanesulfonate EMS treatment) which generates frequent point mutations and small deletions, HII generates a relatively high frequency of large (whole gene) deletion mutations. However, the difficulty of detecting mutants that contain deletions of homoeologues of target genes has been a major factor limiting the application of this method to polyploid species.

Bread wheat (*Triticum aestivum *L.) is one of the most important food crops in the world and is hexaploid, comprising the A, B and D genomes with theoretically all genes present on each of the three homoeologous genomes [19]. Similar to other plants, availability of genetic mutants that facilitate understanding of gene function is highly desirable in wheat. In addition, new genetic variation in mutants can be exploited in traditional plant breeding [6,11]. In wheat, well-recognised examples of the types of genes that are candidates for mutational inactivation are those that encode allergenic and nutritionally poorly tolerated grain proteins [19] as well as grain quality genes such as those that influence starch composition [20]. An emerging new category of genes for which null mutations will be sought are disease susceptibility genes [21-23]. It has become apparent that pathogens often exploit essential cellular processes of their hosts for their own benefit during infection and that multiple host genes may be required to permit a susceptible infection [22]. We have recently demonstrated that inactivation of the *PFT1 *gene of *Arabidopsis *led to increased resistance to the fungal pathogen *Fusarium oxysporum *[23]. The *PFT1 *gene was initially identified as a positive regulator of shade avoidance [24], and subsequently shown to encode a subunit of the plant Mediator complex - a conserved multi-protein complex involved in the fine-tuning of gene expression in all eukaryotes [25]. We are interested to test whether the inactivation of orthologues of *PFT1 *in wheat would have a similar effect on fungal disease resistance. The *PFT1 *gene is highly suited for reverse genetic analysis and the development of a new screening method, because it is a single copy gene in the genomes of diploid species such as Arabidopsis and rice, and a full-length cDNA sequence from wheat is available [23].

In the present study, we have developed a TaqMan single nucleotide polymorphism (SNP) detection method suitable for high-throughput screening of a HII population of hexaploid wheat to identify homoeologous deletion mutants. This method relies upon uniquely fluoro-labelled homoeologue-specific TaqMan SNP-detection probes to identify the presence or absence of homoeologous copies of a gene of interest. Although the TaqMan SNP-detection PCR system has been widely used as a high-throughput method for allelic discrimination in humans [26,27], this method has not been used extensively in plants, with some limited application to the differentiation of SNPs in genes that confer herbicide tolerance [28,29]. The application of TaqMan probes to screening for gene deletions in *Drosophila *has been demonstrated [30]. However, the methodology used in that study differed significantly to the method presented here as gene specific primer/probe combinations with generic probe fluoro-labelling were used in *Drosophila *to assess the presence of diploid loci by comparing fluorescence from individual gene specific qPCRs on the same DNA samples.

Here we describe in detail the design, optimization, validation, and application of a new method for the identification of deletions for each of the three homoeologues of a wheat gene, using *TaPFT1 *as a model. Using this method we have subsequently identified multiple deletion mutants for three additional wheat genes putatively involved in plant defence.

## Results

### Identification of *TaPFT1 *homoeologues in wheat

**Figure 1:**
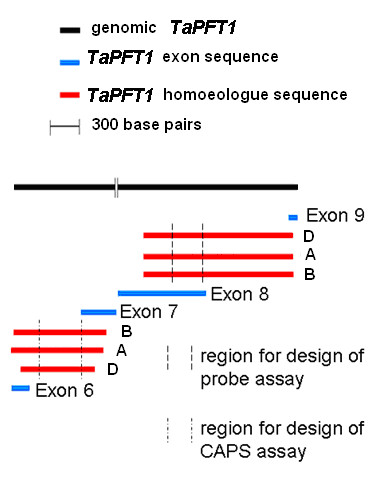
**Overview of *TaPFT1 *sequence obtained and regions used for probe and CAPS assays**. Diagrammatic overview of the partial sequences obtained for wheat *TaPFT1 *homoeologues in regions used in design of the probe-based and CAPS validation assays. Putative intron region between putative exons 7 and 8 (based on alignment to *OsPFT1*) was not sequenced as indicated by the break in the black genomic sequence line.

Based on an existing full-length cDNA sequence for a wheat orthologue (UniGene Ta.39294) [16] of the Arabidopsis *PFT1 *gene in conjunction with the conserved intron-exon structure that exists in the orthologous single copy rice gene for *PFT1 *(Os09g13610), it was possible to design PCR primers (see Materials and Methods) that would generically amplify overlapping fragments of all homoeologous copies of *TaPFT1*. Multiple (30-60) DNA fragments amplified with conserved *TaPFT1 *primers from the wheat cultivar Chara were cloned and sequenced. This resulted in the identification of five unique contigs (designated *TaPFT1a - TaPFT1e*; see Methods) putatively corresponding to sequences that partially spanned between exon 4 and exon 15 of the 15 predicted exons of three *TaPFT1 *homoeologues (Additional file 1). The consensus exon-intron structure of the *TaPFT1 *gene in regions used for the design of the TaqMan probe assay and a Cleaved Amplified Polymorphic Sequence (CAPS) assay for validation are depicted diagrammatically in Figure 1. Sequences corresponding to *TaPFT1 *homoeologues in Chara were deposited into the NCBI database (see Methods for accession numbers). The total polymorphism amongst *TaPFT1 *homoeologous sequences is given in Table 1.

**Table 1:** Sequence diversity identified amongst the three homoeologues of TaPFT1 in the sequenced regions

	base pairs	SNPs	indels	all polymorphism	SNPs/100 bp	indels/100 bp	all poly/100 bp
**exon sequence**	1126	21	8	29	1.9	0.7	2.6

**intron sequence**	4368	214	51	265	4.9	1.2	6.1

**total sequence**	5494	235	59	294	4.2	1.1	5.4

### Probe-Based Detection of *TaPFT1 *Homoeologues

**Figure 2:**
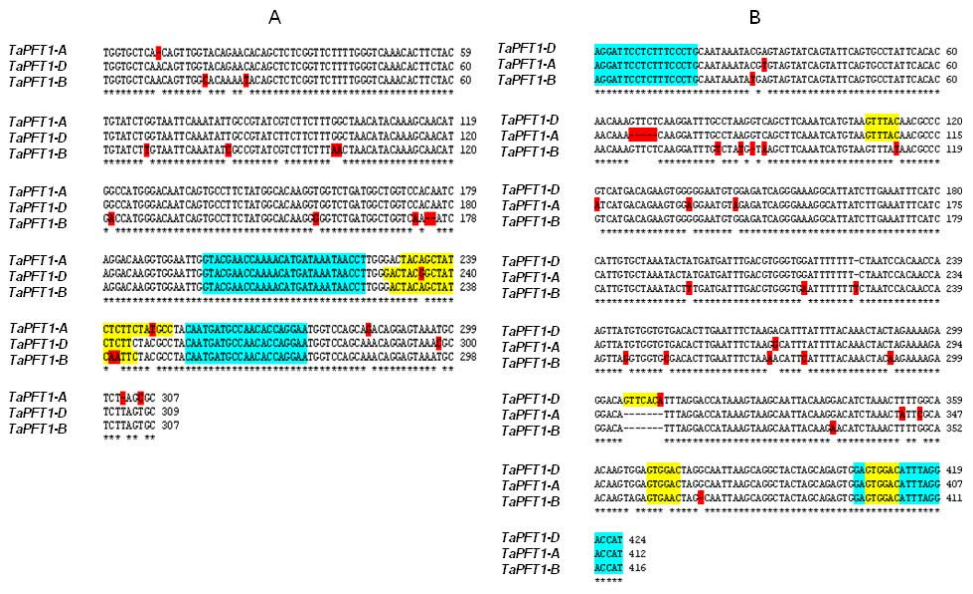
**Alignment of *TaPFT1 *homoeologues**. 'A', 'B' and 'D' *TaPFT1 *homoeologue sequences in regions used for design of the probe assay (A) and the CAPS assay (B), respectively (see Figure 1) were aligned. Forward and reverse primers used in the probe assay are highlighted in blue. Polymorphisms are highlighted in red. In (A), homoeologue specific probe sequences and in (B), restriction enzyme (*Hpy*166II) cleavage sites are highlighted in yellow.

**Figure 3:**
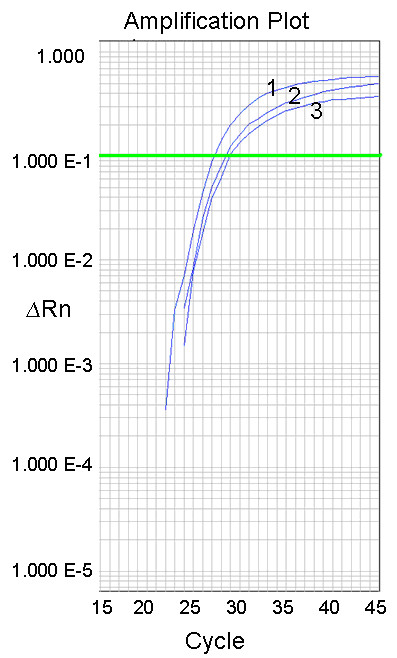
**Amplification profiles of *TaPFT1 *homoeologues in the wheat genotype Chara**. Fluorescence generated from a multiplexed-assay using the *TaPFT1 *PCR primers and homoeologue-specific probes shown in Figure 2A using cv. Chara genomic DNA as a template. A strong increase in fluorescence was detected in the three specific channels each detecting a different fluorescent signal (FAM [curve 1], NED [curve 2], and VIC [curve 3]), from the amplification of *TaPFT1 *homoeologous sequences with C_T _values of approximately 25 for all probes. Data generated in an ABI 7900HT as an absolute quantitation amplification plot. Note that Y axis is a logarithmic scale.

The high-throughput technique we describe here for reliable amplification of *TaPFT1 *homoeologues from wheat is modified from a dual-labelled probe SNP detection assay originally reported by Livak [26]. Based on sequences obtained for putative *TaPFT1 *homoeologues, a region of *TaPFT1 *suitable for the design of short fluorescent hybridisation probes that would distinguish different *TaPFT1 *homoelogues was identified (Figure 1) and three homoeologue specific TaqMan probes labelled with the fluorescent dyes FAM, NED and VIC were designed and synthesised. These probes were then used in qPCR together with a pair of conserved flanking PCR primers that would simultaneously amplify all three *TaPFT1 *homoeologues (Figure 2A). As shown in Figure 3, this assay successfully amplified *TaPFT1 *homoeologues from the genomic DNA isolated from Chara.

### Validation of Assay

**Figure 4:**
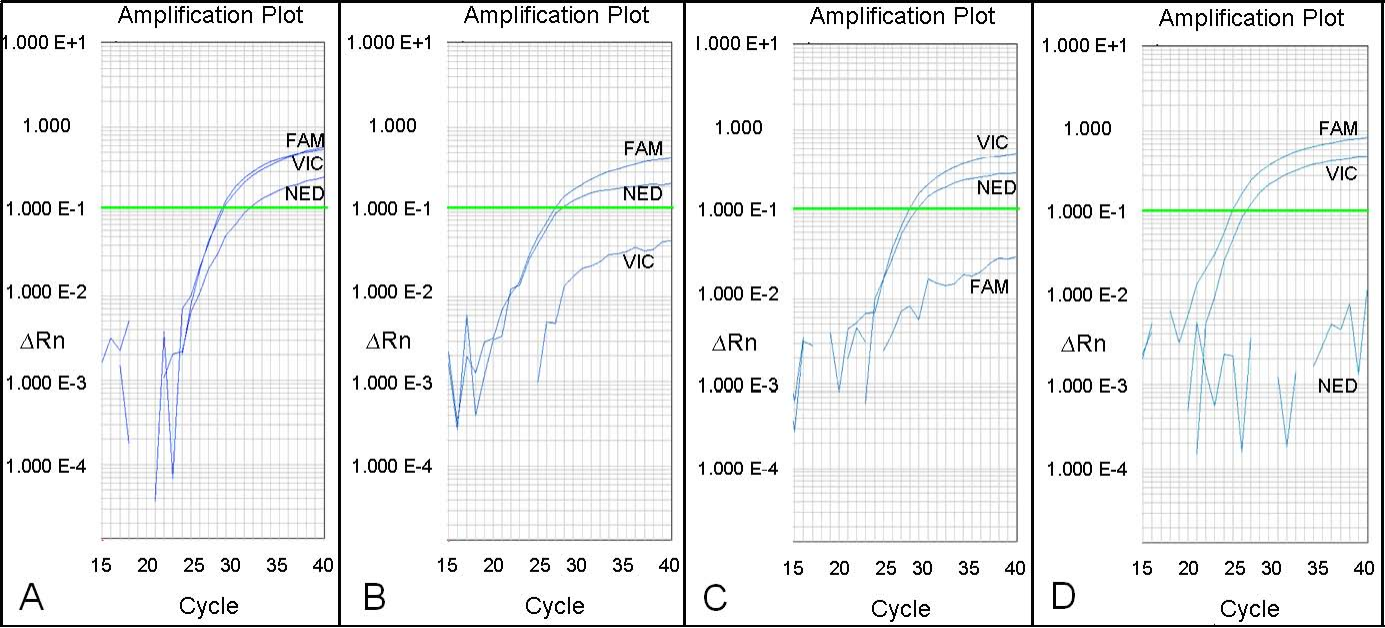
**Amplification profiles of *TaPFT1 *homoeologues in Chinese Spring nullisomic tetrasomic lines**. Fluorescent probe assay on 'wild type' Chinese Spring (A) and samples of Chinese Spring nullisomic tetrasomic wheat lines known to be deficient for chromosomes 5A(B), 5B(C) and 5D(D). A: Reaction features strong, efficient fluorescence from all probes. B: Reaction features strong efficient fluorescence from FAM-labelled (*TaPFT1-B *specific), and NED-labelled (*TaPFT1-D *specific) probes, but very inefficient fluorescence from the VIC-labelled (*TaPFT1-A *specific) probe. C: Reaction features strong efficient fluorescence from VIC-labelled (*TaPFT1-A *specific), and NED-labelled (*TaPFT1-D *specific) probes, but very inefficient fluorescence from the FAM-labelled (*TaPFT1-B *specific) probe. D: Reaction features strong efficient fluorescence from VIC-labelled (*TaPFT1-A *specific), and FAM-labelled (*TaPFT1-B *specific) probes, but very inefficient fluorescence from the NED-labelled (*TaPFT1-D *specific) probe.

**Figure 5:**
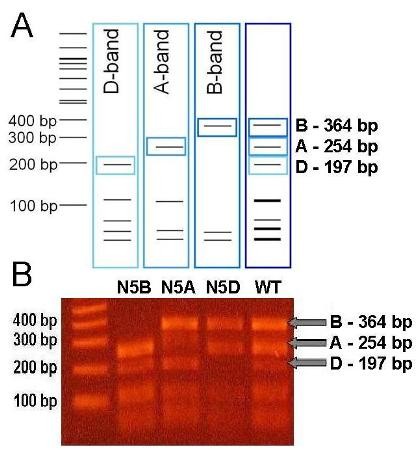
***TaPFT1 *CAPS assay for validation**. **(**A) Predicted CAPS digestion patterns associated with *TaPFT1-D*, *TaPFT1-A *and *TaPFT1-B*. Diagnostic fragments expected upon digestion with *Hpy*166II for *TaPFT1-D *(D-band), *TaPFT1-A *(A-band) and *TaPFT1-B *(B-band) are highlighted in blue boxes. Right-most panel shows the predicted restriction digestion pattern of a sample possessing all *TaPFT1 *homoeologues. (B) CAPS gel analysis and verification of deletion of homoeologue-specific bands from nullisomic-tetrasomic lines N5A, N5B, N5D, and Chinese Spring.

Before an extensive screening of mutagenized wheat populations was undertaken for identification of *TaPFT1 *deletion mutants, we hoped to validate the specificity of this assay by determining the chromosomal location of *TaPFT1 *using genetically well-characterized wheat lines (data not shown). We obtained a series of nullisomic-tetrasomic wheat lines, each missing a single chromosome (nullisomic) substituted by an additional copy of a homoeologous chromosome (tetrasomic) [31,32] as well as a comprehensive smaller deletion series of wheat lines [33]. These lines were generated using the cultivar 'Chinese Spring'. The probe assay functioned successfully against wild type 'Chinese Spring' (Figure 4A), indicating that sequence in the targeted region was conserved between 'Chara' and 'Chinese Spring'. This was confirmed *in silico *by performing a BLAST search of 5× Chinese Spring Whole Genome Sequence 454 raw reads (available at http://www.cerealsdb.uk.net) with *TaPFT1 *sequence obtained from Chara - reads with identical sequence to each of the homoeologous sequences in the region of assay design were detected. As shown in Figure 4, wheat lines individually missing chromosomes 5A, 5B and 5D lacked fluorescence from VIC-, FAM- and NED-labelled probes, respectively. This indicated that *TaPFT1 *was located on chromosome 5. The location of *TaPFT1 *on homoeologous chromosomes 5A, 5B, and 5D, respectively, was confirmed independently using the *TaPFT1 *CAPS screen (Figure 5A and 5B), which was also designed based on the obtained 'Chara' sequence (see Materials and Methods), but shown to function successfully for 'Chinese Spring' samples, as confirmed by BLAST searches at http://www.cerealsdb.uk.net.

To more specifically determine the genomic location of *TaPFT1 *on chromosome 5, we screened additional deletion lines that contain smaller deletions on homoeologous chromosome 5A [33]. These experiments revealed that one line, 5AL-14, lacked the A genome homoeologue of *TaPFT1 *designated here as *TaPFT1-A*. 5AL-14 was the largest long-arm chromosome 5 deletion available in the series and the absence of the amplification of *TaPFT1-A *indicated that this gene was located between the break point of 5AL-14 (FL: 0.11) and the break point of the next largest chromosome 5 deletion, 5AL-12 (FL: 0.35) [33]. Together, these experiments also confirmed that the assay was able to reliably detect presence and/or absence of *TaPFT1 *homoeologues in wheat.

### Identification of *TaPFT1 *deletion mutants

**Figure 6:**
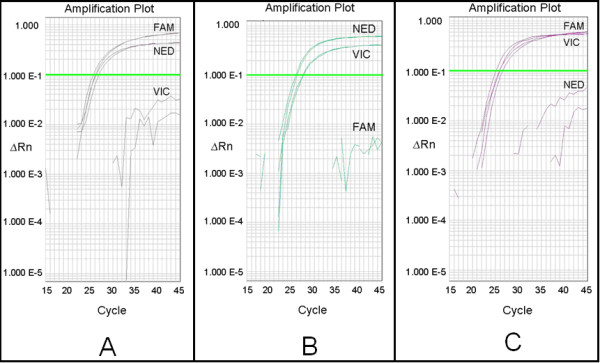
**Amplification profiles of *TaPFT1 *homoeologues in HII mutants lacking one of the *TaPFT1 *homoeologous**. Identification of mutants in homoeologous copies of the *TaPFT1 *gene in the wheat cultivar Chara using the TaqMan fluorescent probe method. (A) Strong fluorescence from FAM-labelled (*TaPFT1-B *specific) and NED-labelled (*TaPFT1-D *specific) probes, but very weak fluorescence from the VIC-labelled (*TaPFT1-A *specific) probe. (B) Strong fluorescence from VIC-labelled (*TaPFT1-A *specific), and NED-labelled (*TaPFT1-D *specific) probes but very weak fluorescence from the FAM-labelled (*TaPFT1-B *specific) probe. (C) Strong fluorescence from VIC-labelled (*TaPFT1-A *specific), and FAM-labelled (*TaPFT1-B *specific) probes, but very weak fluorescence from the NED-labelled (*TaPFT1-D *specific) probe.

**Figure 7:**
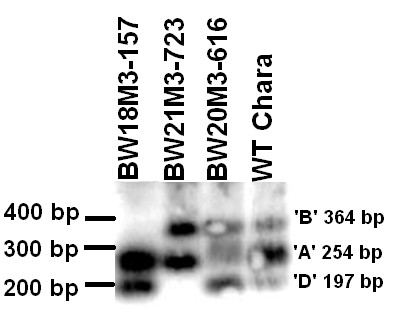
**Verification of homoeologue deletions in *TaPFT1 *HII Chara mutants by CAPS and hybridisation analysis**. A Southern blot prepared from a CAPS assay and hybridised to a *TaPFT1*-specific radiolabelled DNA probe for verification of HII mutants detected by screening using the TaqMan SNP-detection probe-based method. BW18M3-157 (null *TaPFT1-B*); BW21M3-723 (null *TaPFT1-D*); BW20M3-616 (null *TaPFT1-A*) and Chara.

We next applied this assay for the identification of HII mutants that lack at least one of the *TaPFT1 *homoeologues. From the screening of approximately 4500 individual HII mutant (M2 generation) lines of Chara, nine 'definite' mutants were detected, with an additional two 'possible' mutants identified. Mutants were considered 'definite' if efficient fluorescence increases for two of the three probes, with C_T_s (cycle thresholds) below 30 was observed, in conjunction with very low or no fluorescence increase for the third probe for both replicates. Mutants were considered 'possible' if one replicate failed, or if fluorescence from probes had a C_T _value greater than 35, indicative of very low DNA concentration in the sample. Of the screened mutants, five samples (BW18M3-444, BW19M3-132, BW20M3-616, BW22M3-85, and BW22M3-127) lacked fluorescence from VIC-labelled probe 2, indicating the absence of *TaPFT1-A *(Figure 6A); two samples (BW18M3-157 and BW19M3-146) lacked fluorescence from FAM-labelled probe 1, indicating the absence of *TaPFT1-B *(Figure 6B); and four samples (BW19M3-325, BW21M3-723, BW21M3-734 and BW19M3-757) lacked fluorescence from NED-labelled probe 3, indicating the absence of *TaPFT1-D *(Figure 6C). Of the eleven putative mutants, seed stocks were not available for BW18M3-444 and BW19M3-132; and seeds of BW19M3-146 failed to produce viable seedlings. DNA extracted from the remaining lines were screened using the probe-based screen in conjunction with the *TaPFT1 *CAPS screen and in this case, CAPS gels were also blotted and hybridised to a *TaPFT1 *probe to ensure specificity (Figure 7). The results of CAPS screening and hybridisation (Figure 7) demonstrated that genome-specific bands present in Chara were missing in the designated mutants for samples even including the ones that were considered possible in the initial screening.

### Application of method to identification of deletions in other target genes

To confirm that the method described here performs routinely, we have subsequently chosen three additional wheat genes putatively encoding negative regulators of plant disease resistance and screened our HII mutant population to identify homoeologue deletions in these genes. The genes selected were: *TaNFXL1*, *TaCesA8*, and *TaPLDB1*. *TaNFXL1 *is the orthologue of the Arabidopsis *AtNFXL1 *gene demonstrated to be a negative regulator of salicylic acid (SA) accumulation and SA-dependent defence genes. Arabidopsis mutants lacking *AtNFXL1 *show increased resistance to the compatible pathogen *Pseudomonas syringae *pv. *tomato *strain DC3000 (*Pst *DC3000) [34]. *TaCesA8 *is the orthologue of the Arabidopsis *CesA8 *gene shown to encode a susceptibility factor for the bacterial pathogen *Ralstonia solanacearum *and the necrotrophic fungus *Plectosphaerella cucumerina*. Resistance in *cesa8 *mutants is believed to be conferred via an increase in ABA-dependent defence gene expression, and alteration of cell wall structure, leading to the activation of novel defence pathways [35]. *TaPLDβ1 *is the orthologue of *OsPLDβ1*, a rice phospholipase D gene demonstrated to act as a negative regulator of resistance to *Magnaporthe grisea *and *Xanthomonas oryzae *pv *oryzae*. Knockdown of *OsPLDβ1 *results in induction of a range of defence related genes in rice [36].

cDNA sequences corresponding to putative wheat orthologues of these additional target genes were identified using Arabidopsis and/or rice protein sequences as queries in tblastn searches of the NCBI wheat EST sequence database. Sequences for rice orthologues of *AtNFXL1 *and *AtCesA8 *were obtained by performing a tblastn search against the NCBI rice cDNA sequence database. Full genomic sequences are available for all annotated rice cDNAs. Therefore, putative intron/exon structure of corresponding wheat genes was determined by aligning wheat cDNA sequences to rice genomic sequences for all genes. Based on these alignments PCR primers were designed. PCR products were cloned and more than 30 individual clones were sequenced for each gene and, based on these sequences, the three putative homoeologous sequences were deduced. Using these homoeologous sequences, assays were designed as for *TaPFT1 *and applied to the screening of the HII wheat lines. Consequently, 14, 19 and 11 deletion mutants were identified for *TaNFXL1, TaCesA8*, and *TaPLDβ1*, respectively, demonstrating that the assay is broadly applicable to deletion screening in wheat. Details of the design and application of these assays are provided in Table 2.

**Table 2:** Details of the application of the method to the screening of additional candidate genes

gene name	sequence length (bp)^1^	GenBank accessions	primer sequences^2 ^; amplicon size^1^	probe sequences^2^	assay conditions^3^	samples screened; mutations detected
*TaNFXL1*	~630	HQ595068 - HQ595070	**FNFXL1gsprb** GGCACACCAACTCCATCATG **RNFXL1gsprb** GGCTGGTTGCATCTGATATCCT **Amplicon size: **~170 bp	**NFXL1prb1FAM** AACACATTCTCTTGTGACTG **NFXL1prb2VIC** AACGCACTCTCTCATGA **NFXL1prb3NED** ACATTCTCTCATTACTGACACA	**FR primers: **1000 nM each **Probes: **50 nM FAM, 100 nM VIC, 100 nM NED **20 μL reaction: **18 μL stock; 2 μL DNA sample **3-step reaction: **95°C 15 s denaturation, 55°C 30 s annealing, 60°C 30 s extension - 45 cycles	2772; 14

*TaCesA8*	~370	HQ595065 - HQ595067	**FCesA8gsprb** CCACATGGCGAAGAACACCT **RCesA8gsprb** AGTGGACGACGGTGCTGATC **Amplicon size: **~140 bp	**CesA8prb1FAM**CTCATACCCGCTATTGA **CesA8prb2VIC**CCGACCAGGTTGAG **CesA8prb3NED** ACGAGGTTGAGAACC	**FR primers: **1000 nM each **Probes: **50 nM FAM, 100 nM VIC, 100 nM NED **20 μL reaction: **18 μL stock; 2 μL DNA sample **3-step reaction: **95°C 15 s denaturation, 55°C 30 s annealing, 60°C 30 s extension - 45 cycles	3762; 19

*TaPLDβ1*	~620	HQ595071 - HQ595073	**FPLDB1gsprb** TTTGTACTTGTCTTCCTGATGTTATGC **R1PLDB1gsprb** GGCGTAGTTGGAGGCAGTCTC **R2.3PLDB1gsprb** GGTGTAGTTGGAGGCAGTCTCTC **Amplicon size: **~100 bp	**PLDB1prb1FAM** TTGCTTAGAACTGCTGC **PLDB1prb2NED** CTTAGAACCGCTGCTT **PLDB1prb3VIC** TAGAACCACTGCTTC	**FR primers: **1000 nM F primer, 666 nM R2.3 primer, 333 nM R1 primer **Probes: **50 nM FAM, 100 nM VIC, 150 nM NED **11 μL reaction: **9 μL stock; 2 μL DNA sample **3-step reaction: **95°C 15 s denaturation, 55°C 30 s annealing, 60°C 30 s extension - 45 cycles	3366; 11

^1^Approximate; some variation between homoeologues was present ^2^All primer and probe sequences are given in 5' - 3' directions ^3^All assays run in 'Standard Mode' on an ABI 7900HT (see Materials and Methods); all thermal cycling commences with 50°C 2 mins, 95°C 10 mins

## Discussion

A greater understanding of the genetics underlying the phenotypic properties of crop species has improved our ability to manipulate genes responsible for unwanted characteristics. Genes encoding certain grain proteins which can be allergenic or nutritionally poorly tolerated are well recognised examples of genes with unwanted characteristics [19]. Many desirable characteristics are also known to be controlled by recessively inherited loci [37-39] some of which have been shown to be non-functional alleles [40,41]. Furthermore, in the last decade 'negative regulators' of plant resistance have been reported. Loss-of-function mutants of these genes show increased resistance to specific pathogens [21-23]. It therefore seems likely that reverse genetics approaches that exploit the use of gene deletions in crop improvement will increase, especially whilst GM approaches for specific gene inhibition may be restricted in commercial use due to prohibitive legislation in many parts of the world.

In this article we outline a TaqMan SNP-detection probe-based assay optimized for high-throughput screening to identify whole-gene deletion mutants in wheat and other polyploid species. The following strategy was followed to develop and validate the assay. First, sequence analysis was undertaken on the *TaPFT1 *gene to identify homoeologous sequences. This sequence information was then used to design a qPCR assay using specific TaqMan SNP-detection probes that would discriminate the three *TaPFT1 *homoeologues in a single reaction. To confirm the ability of the assay to detect wheat lines missing a specific *TaPFT1 *homoeologue, it was applied to the screening of nullisomic-tetrasomic wheat lines [31,32]. Chromosomal locations of *TaPFT1 *homoeologues were thereby identified, and validated using an independent CAPS-based assay. Finally, automated qPCR screening of a wheat HII population to identify mutants for each *TaPFT1 *homoeologue was performed and identified mutants validated using the CAPS-based assay. After developing and validating this method using *TaPFT1*, we applied it to the detection of homoeologue deletions of three additional target genes (*TaNFXL1*, *TaCesA8 *and *TaPLDβ1*) and successfully identified multiple deletions for each within the HII population (Table 2).

We used patented Applied Biosystems MGB-NFQ TaqMan SNP-detection probes for all assays (see Materials and Methods). We chose this supplier because ABI provided a flexible custom probe design service and assistance with the design of these assays to our specific criteria. However, a range of other SNP-detection fluorogenic qPCR probes are available from other suppliers, and these may also be adaptable to this assay.

As outlined above, using the assay developed we were able to determine the chromosomal location of *TaPFT1-A *by screening nullisomic-tetrasomic lines of the wheat genotype Chinese Spring. We subsequently screened Chinese Spring deletion lines to narrow down its genomic location to a region between FL 0.11 and FL 0.35 on chromosome 5A, close to the centromere region (data not shown). Similarly, using nullisomic-tetrasomic lines of Chinese Spring, *TaPFT1-B *and *TaPFT1-D *were shown to reside on chromosomes 5B and 5D, respectively. Lines with 5B and 5D chromosome deletions that are large enough to exclude these loci based on the location of *TaPFT1-A *were not available, and all screened 5B and 5D deletion lines possessed the respective *TaPFT1 *homoeologues. However, from the results of the nullisomic-tetrasomic lines in conjunction with the largest 5B and 5D long and short arm deletions screened, we could putatively assign *TaPFT1-B *to a location between 5BS FL 0.13 and 5BL FL 0.52; and *TaPFT1-D *to between 5DS FL 0.22 and 5DL FL 0.60, and these locations are consistent with the location determined for *TaPFT1-A*. However, since none of the smaller 5B and 5D deletion lines screened lacked the respective copies, these finer chromosomal locations of *TaPFT1-B *and *TaPFT1-D *will require further definition in the future.

The location of *TaPFT1 *homoeologues on their respective homoeologous chromosomes was confirmed using a *TaPFT1 *CAPS assay (Figure 5B) designed for validation of the probe-based assay. The chromosomal location of *TaPFT1 *in wheat is consistent with the demonstrated location of *OsPFT1 *on chromosome 9 (LOC_Os09g13610 within the 'Rice Genome Annotation Project') which is syntenic to wheat chromosome 5 [42]. Additionally, blasting *TaPFT1 *against the Phytozome v5.0 database http://www.phytozome.net has revealed that putative *Sorghum bicolor SbPFT1 *and *Brachypodium distachyon BsPFT1 *are located on chromosomes 2 and 4, respectively - *Sorghum *chromosome 2 and *Brachypodium *chromosome 4 are indeed syntenic to wheat chromosome 5 [42,43]. However, our results assigning *TaPFT1 *on wheat chromosome 5 conflict with a location of a 554 bp putative *TaPFT1 *EST (accession no. BF472999), which shows approximately 97% homology to *TaPFT1 *sequence that we obtained, on chromosome 7DS in the GrainGenes 2.0 database http://wheat.pw.usda.gov/GG2/index.shtml. One possible explanation for this discrepancy might be the existence of a duplicate *TaPFT1 *locus. However, the results of our sequencing of *TaPFT1 *clearly showed the presence of only three distinct sequences in the hexaploid wheat genotype we used, arguing against the presence of such a duplicate locus. Another possibility could be that a translocation of a *TaPFT1 *locus has occurred in the specific genotype used in mapping. Another, more probable, explanation is that this EST has been incorrectly mapped in the GrainGenes database.

Prior to the development of this technique, screening of the HII lines used in this study for homoeologue deletions of a number of candidate genes had been performed using CAPS assays similar to that performed in this study for validation purposes. The key disadvantages of CAPS screening include: difficulty in identifying sufficient polymorphism for design of the assay, and high labour and time costs of performing large-scale screening. By exploiting probes designed to effectively discriminate between sequences differing by as little as a single base pair, this new assay requires only the most minimal polymorphism between homoeologues. The minimum requirement for successful design of the specific probes are the presence of individual SNPs specific for each homoeologue occurring within a region of suitable length for a qPCR amplicon (e.g. <400 base pairs), that can be amplified from each homoeologue in a single reaction. Where possible, a single primer pair has been used for an individual assay. However, polymorphism in an otherwise desirable site for primer design can be accommodated via the multiplexing of primers showing identity to each of the polymorphic sequences, as for the *TaPLDβ1 *assay (Table 2). With our previous screening using CAPS, a single researcher could process approximately 200 samples per day. In contrast, this new assay allows us to screen up to 384 individual samples (192 individual samples in duplicate) in approximately 2 hours. The time taken to perform the qPCR assay is the limiting factor for screening - a robotic set up of individual plates takes approximately one hour. Using our equipment that allows pre-prepared optical plates to be stacked and continuously loaded and analysed, this theoretically allows for the processing of approximately twelve 384-well plates, corresponding to up to approximately 2000 duplicated samples, in a 24 hour period, in an automated manner, by a single researcher.

It should be noted that, as for genome-specific PCR and CAPS-based deletion screening techniques, this assay is not suitable for the detection of hemizygous individuals (those in which a specific locus is missing from one homologous chromosome but intact within the other) and is not amenable to sample pooling due to its reliance on detection of the lack of specific target sequence. Although the ability to detect individuals hemizygous for a target locus would be advantageous, the accurate identification of hemizygous lines is inherently challenging as the target sequence remains present in these lines, albeit at half the concentration. Whilst it is theoretically possible to discriminate copy number of a target locus using qPCR, we have found that when dealing with variation in DNA sample concentration and quality that can be present in any large collection of samples such as those screened here, hemizygous individuals may not be identified with confidence using this method.

We chose to screen samples in duplicate to provide additional validation of putative mutants, and to cater for failure of a single reaction due to technical error. During the process of screening for homoeologous deletions of the four target genes, we have assessed approximate frequencies of conflicting screening results between duplicate reactions. Technical errors leading to one successful and one failed reaction are indeed very low (< 1 per 96 qPCR reactions); whilst mutant/non-mutant fluorescence profiles, indicative of false positive or negative results, are absent where C_T _values are < 30 and very rare for DNA samples with low concentrations yielding C_T _values >35 (< 1 per 384 qPCR reactions). Therefore, duplication for deletion screening using this method is arguably unnecessary, especially since downstream validation of indexed seed will be required for any identified mutant lines. By eliminating duplication, throughput of this method could be doubled, and cost per sample halved.

Another technology that could be potentially explored for deletion screening in polyploid plant species is automated single-strand conformation polymorphism analysis (SSCP)-based capillary electrophoresis. Theoretically, this technology could allow for the detection of homoeologue-specific target gene deletions. However, the logistics of such an application for this method are unknown, and a recent protocol for mutation screening using this technique indicates that throughput for sample screening (a maximum of 768 samples over three days) [44] is unlikely to be capable of matching that of the presented method (approximately 6000 duplicated samples in the same time period).

Recently, a modified TILLING protocol for the screening of hexaploid wheat for mutations has been developed [15]. This method appears to be an extremely powerful tool for screening to detect point mutations and small deletions simultaneously within homoeologues of mutagenized wheat lines, such as those generated using EMS [6]. The technique outlined here is not appropriate for the screening of homoeologues for non-specific point mutations and small deletions. Rather, it is useful for detection of presence or absence of homoeologous copies of candidate genes in a mutagenized population within which lines possess frequent whole-gene deletions such as the HII population used in this study. For this purpose, this method has significant advantages over any other currently available technique. In addition, HII lines are considered non-GM; therefore, mutants with beneficial characteristics can be integrated into crop improvement programs. However, as for lines generated using chemical mutagenesis, HII lines can possess 'background' mutations. For reverse genetics approaches to crop improvement using this method in conjunction with a HII population, identified lines with beneficial characteristics can be relatively easily 'purified' by successive rounds of backcrossing and re-screening to remove the majority of such background mutations.

As reported here, our design and optimization of this technique has been performed in hexaploid wheat, which globally is the most significant polyploid crop plant. Like wheat, many important plant species including cotton, canola and potato (tetraploid), triticale and oat (hexaploid), strawberry (octoploid), and sugarcane (5 n-14 n [45]) are polyploids. Therefore, this technique has the potential to be adapted to many other polyploid species. In addition to screening for homoeologues in other polyploid species, this assay would be readily adaptable to the screening for duplicated gene copies or closely-related gene family members within diploid species so long as SNPs that allow the design of gene-specific fluorescent probes exist in the sequences to be identified. Whole-genome sequencing efforts are currently underway in many polyploid and diploid crop plants including wheat. The availability of assembled whole-genome sequence information would facilitate the efficient design of assays for virtually any gene/s using this method.

## Conclusions

Here we present a method for automated, high-throughput screening to detect and distinguish individual homoeologues of any wheat gene. We designed this method for the purpose of identifying homoeologous deletions of target genes within a resource of HII mutagenized wheat lines featuring frequent whole-gene deletions. This method is potentially applicable to the identification of homoeologous copies of a target gene within any polyploid species. It may also have utility for the identification of highly homologous genes (e.g. duplicated genes or gene family members) within diploid species.

## Methods

### Plant material

The commercial hexaploid bread wheat cultivar Chara (AWB Seeds Ltd., Australia) was used for characterisation of target gene sequences and for the isolation of homoeologous deletion mutants in these genes. Heavy ion irradiation of grain of cv. Chara was conducted by the RIKEN RI-beam facility in Japan [46] using a Neon ion beam at 50 Gy. Irradiated grain was germinated and selfed to generate an M2 population comprising approximately 20000 lines and leaf material sampled for DNA extraction. Nullisomic-tetrasomic wheat lines [31,32] and deletion wheat lines [33] derived from the cultivar Chinese Spring were used to determine the chromosomal location of the *TaPFT1 *gene. These were kindly provided by Drs. Evans Lagudah and Chunji Liu of CSIRO Plant Industry and are maintained at the USDA-Agricultural Research Service, University of Missouri, Columbia, MO, USA. All plants were grown under glasshouse conditions in either Canberra or Brisbane, Australia.

### DNA Extraction

DNA was extracted from leaf tissue using a Qiagen DNeasy Plant Mini Kit (Qiagen GmbH) in conjunction with a QIAcube (Qiagen GmbH) apparatus for all samples.

### Design of primers and probes

The Applied Biosystems (Applied Biosystems, Foster City, CA) custom probe design service was used to assist in the design of primers and patented minor-groove-binding, non-fluorescent quencher (MGB-NFQ) TaqMan probes. Homoeologous *TaPFT1 *sequences used in the design of the assay with highlighted primer and probe sequences are shown in Figure 2A. The position of the region within *TaPFT1 *is highlighted in Figure 1. Primer and probe sequences for the *TaNFXL, TaCesA8*, and *TaPLDB1 *assays are given in Table 2.

### Sequencing of target genes

Primers for amplification of genomic regions of *TaPFT1*, *TaNFXL1*, *TaCesA8 *and *TaPLDB1 *were designed using Primer Premier 5.1 (Premier Biosoft International, Palo Alto, CA). PCR products were amplified from genomic Chara DNA using Phusion High-Fidelity DNA polymerase (Finnzymes Oy, Keilaranta 16 A, 02150 Espoo, Finland) with a manufacturer-reported error rate of 4.4 × 10^-7^. PCR products were cloned using a TOPO TA cloning kit (Invitrogen, Carlsbad, CA, USA). Plasmid preparations were produced using a QIAprep Spin Miniprep kit (Qiagen GmbH) in conjunction with a QIAcube (Qiagen GmbH) apparatus. The Australian Genome Research Facility (AGRF) plasmid sequencing service (AGRF, Brisbane, QLD, Australia) was used to obtain cloned fragment sequence using forward and reverse M13 primers. Sequence was analysed using Sequencher Version 4.9 Software (Gene Codes Corporation, Ann Arbor, MI, USA). For *TaPFT1*, sequences labelled *TaPFT1a*(1-3) - *TaPFT1e*(1-3) (see Results) were deposited in GenBank (accession numbers HM561279 - HM561293). Labelling of sequences 1-3 was arbitrary and not indicative of origin in terms of progenitor genome. However, genomic origin of the *TaPFT1b *and *TaPFT1c *sequences used in the design of the probe and CAPS assays respectively have subsequently been determined, as outlined in Figure 1 and 2, by screening of nullisomic-tetrasomic 'Chinese Spring' lines. For *TaNFXL1*, *TaCesA8*, and *TaPLDB1*, sequences labelled 1-3 prefixed with gene name were deposited in GenBank. Again, numerical labelling of sequences was arbitrary for each gene. Accession numbers are given in Table 2.

### Optimization of assay for *TaPFT1*

Primers were tested for PCR specificity using Chara DNA template in conjunction with TaqMan Universal PCR Mastermix (Roche Diagnostics GmbH, Mannheim, Germany). PCR was performed using an Applied Biosystems 9600 thermal cycler apparatus (Applied Biosystems, Foster City, CA). PCR conditions were an initial denaturation step at 95°C for 10 minutes, then 45 cycles of a 15 sec, 95°C denaturation step followed by a 60 sec, 60°C annealing/extension step. 5 μL PCR product was visualized on a 1.4% agarose gel stained with Gel Red (Biotum Inc., Hayward, CA) against 1 μL of Invitrogen 1 kb+ DNA ladder (Invitrogen, Carlsbad, CA, USA). A single band of the desired size was observed.

The multiplexed qPCR assay was optimized using Chara DNA template. Final concentrations of forward and reverse primers remained standard at 1000 nM each. All combinations of final concentrations of 50, 100, and 150 nM for the three probes were assessed. Primer/probe mixes were combined with TaqMan Universal PCR mix (final concentration 1×) and water to produce stocks. 9 μL of stock was added to 1 μL 100 ng/μL Chara DNA, or water as a negative control. Four replicate optimization reactions in conjunction with a negative control were set up for each probe concentration combination. All reactions were set up in a single MicroAmp 384-well optical plate (Applied Biosystems, Foster City, CA) and sealed with a MicroAmp optical adhesive cover (Applied Biosystems). qPCR was performed for these samples using an Applied Biosystems 7900HT qPCR apparatus (Applied Biosystems, Foster City, CA). Thermal cycler was run in '9600 Emulation' mode. Fluorescence detection was in 'Absolute Quantification' mode using three detection channels: Fam-NonFluorescent; Ned-NonFluorescent; and Vic-NonFluorescent. Individual detection channels corresponded to fluorescence produced by individual probes. Optimum fluorescence for all probes in the multiplexed assay was obtained using final concentrations of 50 nM for the 'FAM' and 'VIC' labelled probes and 100 nM for the 'NED' labelled probe. Under these reaction conditions efficient increase of fluorescence from all probes was observed, with C_T_s of approximately 25 cycles for all probes (Figure 3).

In order to assess the specificity of probe fluorescence, 1/10000 dilutions of plasmid preparations, determined by sequencing to harbour cloned PCR products corresponding to regions of homoeologues '1', '2', and '3' targeted by the assay, were prepared. Four replicate reactions for each of five templates: diluted clone A (homoeologue 1), diluted clone B (homoeologue 2), diluted clone C (homoeologue 3), Chara (positive control), and water (negative control) were prepared in a 384-well optical plate. qPCR was performed as above. For all replicates using specific diluted clone templates, efficient fluorescence increase was observed from the corresponding specific probe (i.e. diluted clone 1 template: efficient 'FAM' fluorescence; diluted clone 2 template: efficient 'NED' fluorescence; diluted clone 3 template: efficient VIC fluorescence), but not from the non-specific probes. This indicated that the probe fluorescence was highly specific; non-specific fluorescence was low, or absent, even where non-specific homoeologous sequence was in high concentration and not in competition with the specific homoeologous sequence.

Optimization of assays for other target genes followed the same basic steps as outlined above i.e. 1. assess primer specificity; 2. optimize multiplexed probe assay against Chara DNA template; and 3. assess specificity of probe fluorescence using diluted cloned homoeologue sequence. For the *TaNFXL1 *and *TaCesA8 *assays, reaction volumes were increased to 20 μL (18 μL stock; 2 μL DNA sample) for optimum results. Details of optimized assays are given in Table 2.

### Assessment of limit of detection

To assess the limit of detection of the method in terms of DNA concentration in a sample, serial 1:5 dilutions of a Chara DNA sample were prepared such that resulting samples had concentrations of 91 ng/μL (undiluted); 18.2 ng/μL; 3.7 ng/μL; 0.7 ng/μL; 0.14 ng/μL. 2 μL volumes of these samples were then assayed using the *TaPFT1 *probe-based method, and the *TaPFT1 *CAPS method. Both methods showed consistent results (i.e. the same profiles in three independent reactions) with as little as 0.7 ng/μL DNA concentration.

### High-throughput, automated screening of HII mutant lines to identify individuals lacking homoeologous copies of target genes

The optimized probe method was used for the screening of 2500-4500 individual HII-generated Chara mutant lines per gene in order to identify mutants lacking homoeologues of target genes. DNA from individual M2 mutant lines arranged in indexed 96-well plate format was used for screening. Samples were chosen from a resource of approximately 20000 individual M2 mutant lines (as outlined above). Stock composed of TaqMan Universal Master Mix (Roche), optimized concentrations of primers and probes, and water was combined with template DNA extracted from mutant lines in a 384-well optical plate using an Eppendorf ep*MOTION *5075 apparatus (Eppendorf Australia, Sydney, NSW, Australia). Each individual DNA sample corresponding to an individual M2 line was analysed in duplicate and 192 individual mutant lines were screened per 384-well plate qPCR reaction. Results were assessed using SDS software (Applied Biosystems). Lines identified as lacking a homoeologous gene copy were recorded and M3 seed corresponding to these lines was obtained.

### 'CAPS' screen for validation of mutants detected by *TaPFT1 *probe screen

A region of *TaPFT1 *with relatively high polymorphism between homoeologues, suitable for the design of a CAPS screen [47], was identified from sequence obtained for *TaPFT1 *(Figure 2B). Primers to amplify this region from all homoeologues non-specifically were designed using Primer Premier 5.1 (Premier Biosoft International, Palo Alto, CA). A single restriction enzyme producing clearly distinguishable banding patterns upon cleavage of each homoeologue was identified using the New England Biolabs 'NEBcutter V2.0' application [48] (Figure 5A). The CAPS screen was optimized using Chara DNA. Upon digestion of the amplified product, the expected banding pattern (as predicted by NEBcutter V2.0) was observed. The optimized CAPS screen was performed on Chinese Spring DNA, in conjunction with nullisomic-tetrasomic DNA determined by the probe-screen to lack homoeologous copies of *TaPFT1*. The banding patterns for Chinese Spring, and Null5A, Null 5B and Null 5D (shown to lack the respective homoeologous copies of *TaPFT1*) samples were as predicted by 'Virtual Digest' (Figure 5B). DNA from seedlings corresponding to each specific mutant line identified as lacking a *PFT1 *homoeologue was assessed using the optimized CAPS screen described above.

### Southern blotting of *TaPFT1 *CAPS fragments

DNA fragments were transferred to an Amersham Hybond-XL (GE Healthcare Life Sciences) nylon membrane using the 'Neutral transfer protocol' as outlined in the Amersham Hybond-XL product booklet. Fragments were hybridised with a labelled *TaPFT1 *fragment amplified from cloned *TaPFT1 *fragment 'B' (Figure 1) plasmid preparation using *TaPFT1 *specific forward and reverse primers and radiolabelled with [alpha-^32^P] dCTP using an Amersham Megaprime DNA labelling kit (GE Healthcare Life Sciences). Hybridisation was performed overnight at 65°C and the blot washed in high stringency according to instructions given by the manufacturer. The blot was then exposed to an X-ray film and developed using standard procedures.

## Authors' contributions

ZL and MKM developed the HII wheat mutant population resource and provided DNA samples. TLF designed and optimized the method, performed mutation screening, and assisted in writing the paper. KK and JMM assisted in design and coordination of the project and in writing the paper. All authors read and approved the final manuscript.

## Supplementary Material

Additional file 1 **Figure S1**. Diagrammatic overview of all partial sequences (*TaPFT1a *- *TaPFT1e*) obtained for wheat *TaPFT1 *homoeologues. Click here for file

## References

[B1] Doyle JJ Flagel LE Paterson AH Rapp RA Soltis DE Soltis PS Wendel JF Evolutionary genetics of genome merger and doubling in plants Ann Rev Genet 2008 42 443 461 10.1146/annurev.genet.42.110807.091524 18983261

[B2] Adams KL Cronn R Percifield R Wendel JF Genes duplicated by polyploidy show unequal contributions to the transcriptome and organ-specific reciprocal silencing Proc Nat Acad Sci USA 2003 100 4649 4654 10.1073/pnas.0630618100 12665616 PMC153610

[B3] Hovav R Udall JA Chaudhary B Rapp R Flagel L Wendel JF Partitioned expression of duplicated genes during development and evolution of a single cell in a polyploidy plant Proc Nat Acad Sci USA 2008 105 6191 6195 10.1073/pnas.0711569105 18420816 PMC2329682

[B4] Foley RC Singh KB TGA5 acts as a positive and TGA4 acts as a negative regulator of ocs element activity in Arabidopsis roots in response to defence signals FEBS Lett 2004 563 141 145 10.1016/S0014-5793(04)00288-1 15063738

[B5] Fu D Uauy C Blechl A Dubcovsky J RNA interference for wheat functional gene analysis Transgenic Res 2007 16 689 701 10.1007/s11248-007-9150-7 17952622

[B6] Slade AJ Fuerstenberg SI Loeffler D Steine MN Facciotti A reverse genetic, nontransgenic approach to wheat crop improvement by TILLING Nat Biotechnol 2004 23 75 81 10.1038/nbt1043 15580263

[B7] Shimbata T Nakamura T Vrinten P Saito M Yonemaru J Seto Y Yasuda H Mutations in wheat starch synthase II genes and PCR-based selection of a SGP-1 null line Theor Appl Genet 2005 111 1072 1079 10.1007/s00122-005-0032-1 16172895

[B8] Konik-Rose C Thistleton J Chanvrier H Tan I Halley P Gidley M Kosar-Hashemi B Wang H Larroque O Ikea J McMaugh S Regina A Rahman S Morell M Li Z Effects of starch synthase IIa gene dosage on grain, protein and starch in endosperm of wheat Theor Appl Genet 2007 115 1053 1065 10.1007/s00122-007-0631-0 17721773

[B9] Vrinten P Nakamura T Yamamori M Molecular characterisation of *waxy *mutations in wheat Mol General Genet 1999 261 463 471 10.1007/s004380050989 10323226

[B10] Ajjawi I Lu Y Savage LJ Bell SM Last RL Large scale reverse genetics in *Arabidopsis*: Case studies from the Chloroplast 2010 project Plant Physiol 2010 152 529 540 10.1104/pp.109.148494 19906890 PMC2815874

[B11] Parry MAJ Madgwick PJ Bayon C Tearall K Hernandez-Lopez A Baudo M Rakszegi M Hamada W Al-Yassin A Ouabbou H Labhilili M Phillips AL Mutation discovery for crop improvement J Exp Bot 2009 60 2817 2825 10.1093/jxb/erp189 19516074

[B12] Uauy C Paraiso F Colasuonno P Tran RK Tsai H Berardi S Comai L Dubcovsky J A modified TILLING approach to detect induced mutations in tetraploid and hexaploid wheat BMC Plant Biol 2009 9 115 10.1186/1471-2229-9-115 19712486 PMC2748083

[B13] McCallum C Comai L Greene E Henikoff S Targeting induced local lesions IN genomes (TILLING) for plant functional genomics Plant Physiol 2000 123 439 442 10.1104/pp.123.2.439 10859174 PMC1539256

[B14] Stephenson P Baker D Girin T Perez A Amoah S King G Ostergaard L A rich TILLING resource for studying gene function in *Brassica rapa* BMC Plant Biol 2010 10 62 10.1186/1471-2229-10-62 20380715 PMC2923536

[B15] Dong C Vincent K Sharp P Simultaneous mutation detection of three homoeologous genes in wheat by High Resolution Melting analysis and Mutation Surveyor BMC Plant Biol 2009 9 143 10.1186/1471-2229-9-143 19958559 PMC2794869

[B16] Kazama Y Saito H Yamamoto YY Hayashi Y Ichida H Ryuto H Fukunishi N Abe T LET-dependent effects of heavy-ion beam irradiation in *Arabidopsis thaliana* Plant Biotech 2009 25 113 117

[B17] Honda I Kikuchi K Matsuo S Fukuda M Saito H Ryuto H Fukunishi N Abe T Heavy-ion induced mutants in sweet pepper isolated by M1 plant selection Euphytica 2006 152 61 66 10.1007/s10681-006-9177-5

[B18] Shitsukawa N IKari C Shimada S Kitagawa S Sakamoto K Saito H Ryuto H Fukunishi N Abe T Takumi S Nasuda S Murai K The einkorn wheat (*Triticum monococcum*) mutant, maintained vegetative phase, is caused by a deletion in the *VRN1 *gene Genes Genet Syst 2007 82 167 170 10.1266/ggs.82.167 17507783

[B19] Shewry PR Wheat J Exp Bot 2009 60 1537 1553 10.1093/jxb/erp058 19386614

[B20] Sestili F Botticella E Bedo Z Phillips A Lafiandra D Production of novel allelic variation for genes involved in starch biosynthesis through mutagenesis Mol Breeding 2010 25 145 154 10.1007/s11032-009-9314-7

[B21] Thatcher LF Manners JM Kazan K *Fusarium oxysporum *hijacks COI1-mediated jasmonate signaling to promote disease development in Arabidopsis Plant J 2009 58 927 939 10.1111/j.1365-313X.2009.03831.x 19220788

[B22] Pavan S Jacobsen E Visser RGF Bai Y Loss of susceptibility as a novel breeding strategy for durable and broad spectrum resistance Mol Breeding 2010 25 1 12 10.1007/s11032-009-9323-6 PMC2837247 20234841

[B23] Kidd BN Edgar CI Kumar KK Aitken EA Schenk PM Manners JM Kazan K The mediator complex subunit PFT1 is a key regulator of jasmonate-dependent defense in Arabidopsis Plant Cell 2009 8 2237 2252 10.1105/tpc.109.066910 PMC2751954 19671879

[B24] Cerdán PD Chory J Regulation of flowering time by light quality Nature 2003 423 881 885 10.1038/nature01636 12815435

[B25] Bäckström S Elfving N Nilsson R Wingsle G Björklund S Purification of a plant Mediator from *Arabidopsis thaliana *identifies PFT1 as the Med25 subunit Mol Cell 2007 26 717 729 10.1016/j.molcel.2007.05.007 17560376

[B26] Livak KJ Allelic discrimination using fluorogenic probes and the 5' nuclease assay Genet Anal: Biomol Eng 1999 14 143 149 10.1016/S1050-3862(98)00019-9 10084106

[B27] Latif S Bauer-Sardina I Ranade K Livak KJ Kwok PY Fluorescence polarization in homogeneous nucleic acid analysis II: 5'-Nuclease assay Genome Res 2001 11 436 440 10.1101/gr.156601 11230167 PMC311069

[B28] Giancola S McKhann HI Berard A Camilleri C Durand S Libeau P Roux F Reboud X Gut IG Brunel D Utilisation of the three high-throughput SNP genotyping methods, the GOOD assay, Amplifluor and TaqMan, in diploid and polyploidy plants Theor Appl Genet 2006 112 1115 1124 10.1007/s00122-006-0213-6 16453133

[B29] Warwick SI Xu R Sauder C Beckie HJ Acetolactate synthase target-site mutations and single nucleotide polymorphism genotyping in ALS-resistant Kochia (*Kochia scoparia*) Weed Sci 2008 56 797 806 10.1614/WS-08-045.1

[B30] Chiang P-W Wei W-L Gibson K Bodmer R Kurnit DM A fluorescent quantitative PCR approach to map gene deletions in the Drosophila genome Genetics 1999 153 1313 1316 10545461 10.1093/genetics/153.3.1313 PMC1460813

[B31] Sears ER Riley R, Lewis KR Nullisomic-tetrasomic combinations in hexaploid wheat Chromosome manipulations in plant genetics 1966 Oliver and Boyd, London 29 45

[B32] Sears ER Sears LMS Ramanujam S The telocentric chromosomes of common wheat Proceedings of the 5th International Wheat Genetics Symposium. Indian Society of Genetics and Plant Breeding New Delhi 1978 389 407

[B33] Endo TR Gill BS The deletion stocks of common wheat J Hered 1996 87 295 307

[B34] Asano T Masuda D Yasuda M Nakashita H Kudo T Kimura M Yamaguchi K Nishiuchi T AtNFXL1, an Arabidopsis homologue of the human transcription factor NF-X1, functions as a negative regulator of the trichothecene phytotoxin-induced defense response Plant J 2008 53 450 464 10.1111/j.1365-313X.2007.03353.x 18069941

[B35] Hernandez-Blanco C Feng DX Hu J Sanchez-Vallet A Deslandes L Llorente F Berrocal-Lobo M Keller H Barlet X Sanchez-Rodriguez C Impairment of cellulose synthases required for Arabidopsis secondary cell wall formation enhances disease resistance Plant Cell 2007 19 890 903 10.1105/tpc.106.048058 17351116 PMC1867366

[B36] Yamaguchi T Kuroda M Yamakawa H Ashizawa T Hirayae K Kurimoto L Shinya T Shibuya N Suppression of a phospholipase D gene, OsPLD{beta}1, activates defense responses and increases disease resistance in rice Plant Physiol 2009 150 308 319 10.1104/pp.108.131979 19286937 PMC2675732

[B37] Seversike TM Purcell LC Gbur E Chen P Scott R Radiation interception and yield responses to increased leaflet number in early-maturing soybean genotypes Crop Sci 2009 49 281 289 10.2135/cropsci2007.08.0472

[B38] Kikuchi F Futsuhara Y Matsuo T, Shimizu S, Tsunoda S, Murata Y, Kumazawa K, Futsuhara Y, Hoshikawa K, Yamaguchi H, Kikuchi F Inheritance of morphological characters. 2. Inheritance of semidwarf Science of the Rice Plant 1997 3 Tokyo Food and Agricultural Policy Research Center, Tokyo 309 317

[B39] Cordeiro GM Christopher MJ Henry RJ Reinke RF Identification of microsatellite markers for fragrance in rice by analysis of the rice genome sequence Mol Breeding 2002 9 245 250 10.1023/A:1020350725667

[B40] Bradbury LMT Fitzgerald TL Henry RJ Jin Q Waters DLE The gene for fragrance in rice Plant Biotechnol J 2005 3 363 370 10.1111/j.1467-7652.2005.00131.x 17129318

[B41] Spielmeyer W Ellis MH Chandler PM Semidwarf (sd-1), "green revolution" rice, contains a defective gibberellin 20-oxidase gene Proc Nat Acad Sci USA 2002 99 9043 9048 10.1073/pnas.132266399 12077303 PMC124420

[B42] Bennetzen J Chen M Grass genomic synteny illuminates plant genome function and evolution Rice 2008 1 109 118 10.1007/s12284-008-9015-6

[B43] International Brachypodium Initiative Genome sequencing and analysis of the model grass *Brachypodium distachyon* Nature 2010 463 763 768 10.1038/nature08747 20148030

[B44] Larsen LA Jespersgaard C Andersen PS Single-strand conformation polymorphism analysis using capillary array electrophoresis for large-scale mutation detection Nat Protocols 2007 2 1458 1466 10.1038/nprot.2007.200 17545982

[B45] Burner DM Legendre BL Cytogenetic and fertility characteristics of elite sugarcane clones Sugar Cane 1994 1 6 10

[B46] Ryuto H Fukinishi N Hayashi Y Ichida H Abe T Kase M Yano Y Heavy ion bean irradiation facility for biological samples in RIKEN Plant Biotech 2008 25 119 122

[B47] Konieczny A Ausubel FM A procedure for mapping *Arabidopsis *mutations using co-dominant ecotype-specific PCR-based markers Plant J 1993 4 403 410 10.1046/j.1365-313X.1993.04020403.x 8106085

[B48] Vincze T Posfai J Roberts RJ NEBcutter: a program to cleave DNA with restriction enzymes Nucl Acids Res 2003 31 3688 3691 10.1093/nar/gkg526 12824395 PMC168933

