# Supplementary figures and images for: A high-throughput method for the detection of homoeologous gene deletions in hexaploid wheat
Source: BMC Plant Biol. 2010 Nov 29;10:264. doi: 10.1186/1471-2229-10-264 (PMC3017838; doi:10.1186/1471-2229-10-264)

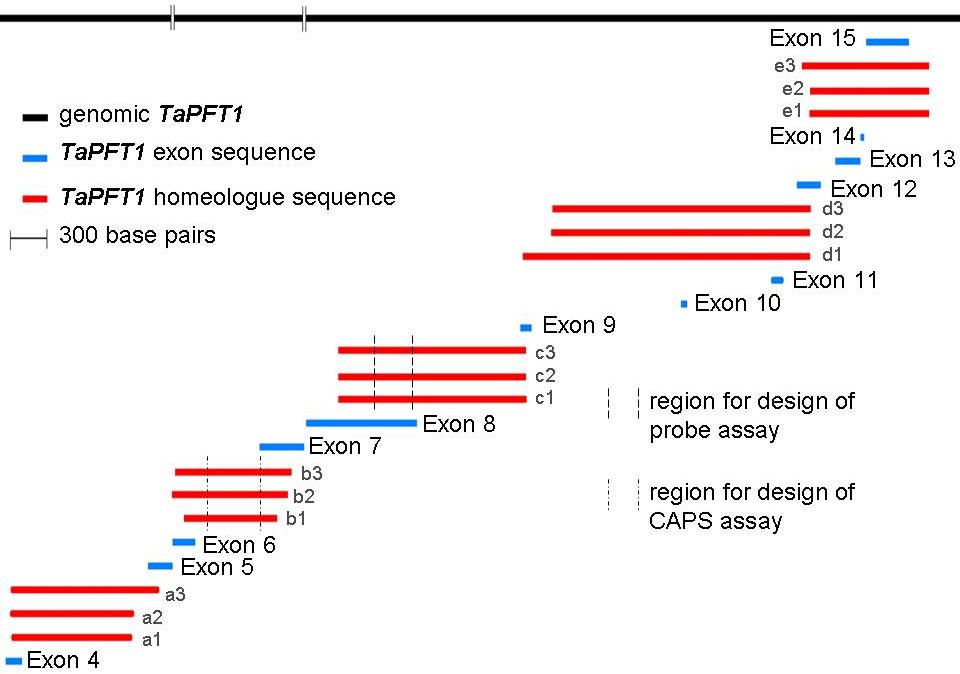

Supplement: Additional file 1 — Figure S1. Diagrammatic overview of all partial sequences (TaPFT1a - TaPFT1e) obtained for wheat TaPFT1 homoeologues. [file 1471-2229-10-264-S1.JPEG]
